# Electrophile Modulation of Inflammation: A Two-Hit Approach

**DOI:** 10.3390/metabo10110453

**Published:** 2020-11-10

**Authors:** James O’Brien, Stacy G. Wendell

**Affiliations:** Department of Pharmacology and Chemical Biology, University of Pittsburgh, Pittsburgh, PA 15261, USA; jpo30@pitt.edu

**Keywords:** immunometabolism, electrophile, inflammation

## Abstract

Electrophilic small molecules have gained significant attention over the last decade in the field of covalent drug discovery. Long recognized as mediators of the inflammatory process, recent evidence suggests that electrophiles may modulate the immune response through the regulation of metabolic networks. These molecules function as pleiotropic signaling mediators capable of reversibly reacting with nucleophilic biomolecules, most notably at reactive cysteines. More specifically, electrophiles target critical cysteines in redox regulatory proteins to activate protective pathways such as the nuclear factor erythroid 2-related factor 2-Kelch-like ECH-associated protein 1 (Nrf2-Keap1) antioxidant signaling pathway while also inhibiting Nuclear Factor κB (NF-κB). During inflammatory states, reactive species broadly alter cell signaling through the oxidation of lipids, amino acids, and nucleic acids, effectively propagating the inflammatory sequence. Subsequent changes in metabolic signaling inform immune cell maturation and effector function. Therapeutic strategies targeting inflammatory pathologies leverage electrophilic drug compounds, in part, because of their documented effect on the redox balance of the cell. With mounting evidence demonstrating the link between redox signaling and metabolism, electrophiles represent ideal therapeutic candidates for the treatment of inflammatory conditions. Through their pleiotropic signaling activity, electrophiles may be used strategically to both directly and indirectly target immune cell metabolism.

## 1. Introduction

Inflammation is essential to the management of both sterile and infectious insults. The cellular processes regulating the resolution of these inflammatory responses are required to prevent unnecessary host damage [1]. The negative sequalae of unattenuated inflammation is clearly illustrated in chronic diseases such as atherosclerosis, rheumatological disorders, and metabolic syndrome, among others [2,3,4]. Under normal conditions, the immune system leverages a number of resolving mechanisms to attenuate host damage [5,6]. More specifically, through adaptive signaling mechanisms, the immune system is capable of both sensing as well as responding to the changing inflammatory environment in order to reach self-limitation [1,7].

During pathological inflammatory states, cells of both the innate and adaptive immune system can create a highly oxidative environment [8]. Reactive oxygen species (ROS), reactive nitrogen species (RNS), and oxides of nitrogen are generated from oxygen through a number of subcellular processes. Mitochondrial ROS (mROS), for example, are produced during the operation of the electron transport chain (ETC) as electrons leak out and partially reduce O_2_ [9]. Other sources of ROS include NADPH oxidases (NOX) required for innate immune cell oxidative burst, myeloperoxidase (MPO) used by neutrophils during anti-bacterial activity, as well as fatty acid oxidation by cyclooxygenases and lipoxygenases [10,11]. 

Reactive species (RS) produced during inflammation can damage the cell. However, recently, studies have suggested the ROS are both a byproduct and regulator of changes in immune cell metabolism required for maturation and effector function [12]. These changes in the redox balance of the activated immune cell are normally accompanied by detoxifying cellular programs and adaptive cell signaling [13]. Several studies have characterized redox regulation in the evolving immune response [14,15]. Interestingly, the production of both RS and lipophilic signaling mediators in the context of inflammation, leads to the production of electrophilic species [16]. 

Formed both enzymatically and non-enzymatically, electrophiles function as key mediators of the inflammatory response [16]. Many of the species formed during inflammation are considered to be “soft-electrophiles,” meaning that they are reversibly reactive with nucleophilic biomolecules. This feature makes electrophiles ideal mediators of cell signaling, informing the cell of the redox balance and extracellular environment. It is well documented that moderate oxidative stress triggers a cellular gene program conferring protection and survival. More specifically, electrophilic species engage the Electrophilic Response Element/Antioxidant Response Element (EpRE)/(ARE) to form downstream defense products [17,18]. Through their activity as pleiotropic signaling mediators and potentiators of antioxidant processes, electrophiles represent an emerging class of anti-inflammatory molecules that promote resolution through a two-hit approach by (1) the direct post-translational modification (PTM) of redox regulatory transcription factors and enzymes that mitigate pro-inflammatory cytokine and chemokine formation and (2) through secondary effects that result in the modulation of immune cell metabolism to dampen the inflammatory response. 

## 2. Electrophile Overview, Structure and Function

**Figure 1:**
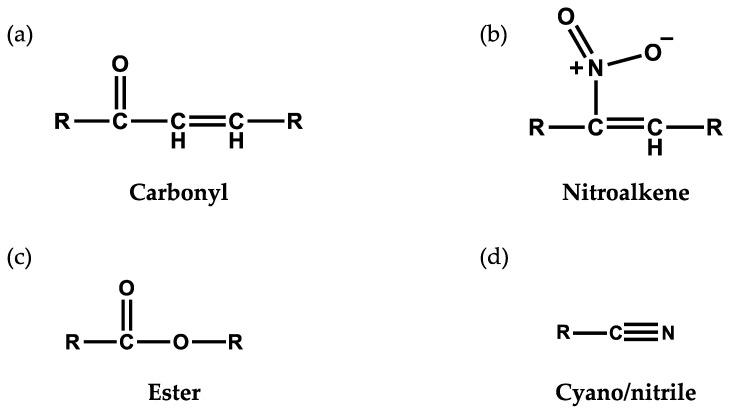
Electrophilic functional groups. These chemical moieties contain an electrophilic carbon that is susceptible to nucleophilic attack from electron-rich biomolecules such as a reactive thiol. Illustrated here are (**a**) carbonyl, (**b**) nitroalkene, (**c**) ester and (**d**) cyano/nitrile functional groups.

Electrophiles are molecules containing one or more electron-poor atoms with an electron withdrawing functional group. Common functional groups capable of creating an active electrophilic carbon include α,β unsaturated carbonyl (R–C_2_H_2_CO–R), ester (–RCO_2_R), cyano/nitrile group (–RCN), or nitroalkene (–RCNO_2_CHR–). These chemical moieties contain a β carbon that is susceptible to a nucleophilic attack from electron-rich biomolecules such as a reactive thiol. The relative reactivity of the electrophile, and subsequent influence on cell signaling, is dictated by the strength of the electron withdrawing groups [19] Figure 1 illustrates a number of electrophilic functional groups. 

These molecules can be formed either enzymatically or non-enzymatically. Enzyme-catalyzed electrophile generation has been found to occur during periods of both redox stress and normal metabolism. This process can be catalyzed by oxygenases and oxidoreductases/dehydrogenases in a multi-step process [20,21]. In contrast, nonenzymatic electrophile generation occurs in oxidizing environments in which ROS and RNS including nitrogen oxides and, lipid alkoxyl/peroxyl radicals potentiate the oxidation and nitration of polyunsaturated fatty acids (PUFAs) [22,23]. Interestingly, in an ischemia reperfusion model, the nitroalkene, nitro-conjugated linoleic acid (NO_2_-cLA), was found to form endogenously in the heart [24]. Electrophilic species formed through lipid peroxidation include malondialdehyde (MDA), 4-hydroxynonenal (HNE), 4-oxononenal (ONE), acrolein, and cyclopentenone neuroprostanes [25,26,27,28,29]. 

As bioactive molecules, electrophilic species can adduct to nucleophilic nucleotides, amino acid residues of proteins, and other small molecules [30,31,32]. In addition to modulating cell signaling events directly through post-translational modification, cellular activity can also be modulated at the level of redox homeostasis as electrophiles interact with the thiol group of glutathione (GSH). Previously published work has demonstrated that electrophiles can alter reducing elements within the cell through both direct sequestration of available GSH equivalents and through the PTM of redox regulatory proteins resulting in the translation of proteins that increase GSH synthesis [33,34,35]. 

**Figure 2:**
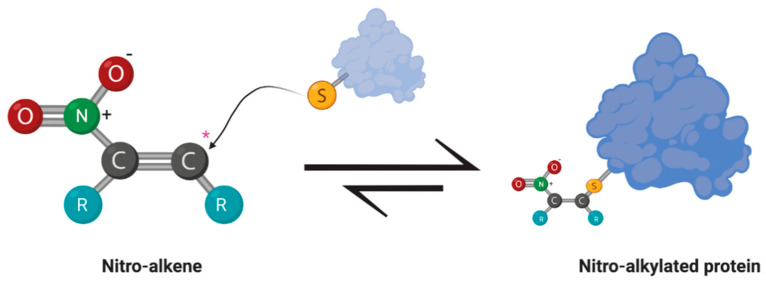
Michael addition between an electrophilic nitroalkene and a nucleophilic thiol group of a protein. The nitro moiety creates an electrophilic carbon (*) susceptible to nucleophilic attack by the thiol group of an amino acid residue, such as cysteine. Reactions with soft electrophiles create a reversible adduct that can alter the cell signaling activity or enzymatic activity of the effected protein.

In the setting of inflammation, electrophiles are endogenously produced and function as cell signaling mediators. Protein functional groups containing nucleophilic moieties are subject to adduction by these electrophilic species. As soft electrophiles, this reaction is reversible, but capable of creating PTM at active cysteine, lysine, and histidine residues, thereby altering enzyme activity or signaling functionality in the cell. A visual representation of a Michael Addition with an electrophilic, nitro-alkene is outlined in Figure 2. While these PTM most commonly occur on cysteine thiols during redox signaling, a Schiff base may also form with amine groups found on lysine and histidine residues. Each electrophile has unique signaling activity based on relative reactivity and structure. As mentioned above, most of these molecules can signal through Keap1-Nrf2 pathway as a part of the EpRE/ARE [17]. The cis-acting element of this cellular program is found in the regulatory region of over 200 genes, the products of which are involved in oxidative and xenobiotic stress responses. Well-known downstream targets of the EpRE/ARE include NAD(P)H Quinone Dehydrogenase 1 (NQO1), Heme Oxygenase-1 (HO-1), Glutamate-Cysteine Ligase (GCL), Gamma-Glutamyl Cysteine Synthetase (γ-GCS), Glutathione-*S*-Transferase (GSTs), and thioredoxins [36,37]. In addition to increasing the expression of genes directly involved in redox homeostasis, electrophiles can alter the redox signaling indirectly through the inhibition of the pro-inflammatory transcription factor, nuclear factor kappa light chain enhancer of activated B cells (NF-κB) [38,39]. NF-κB increases the expression of cyclooxygenase (COX)-2, a pivotal first step in the production of electrophilic α,β-unsaturated ketones, and inducible nitric oxide synthase (iNOS), the enzyme responsible for propagating **^•^**NO production [40,41]. Interestingly, peroxisome proliferator-activated receptor gamma (PPARγ), another downstream target of electrophilic lipids, is a regulatory node for peroxisome proliferation in adipocytes. Peroxisomes function as a subcellular location for fatty acid catabolism and ROS production [42,43,44]. 

There is a biphasic response to electrophile concentration in a cell. For example, at low concentrations, the endogenously formed lipid electrophile, 15-Deoxy-Δ^12,14^-prostaglandin J_2_ (15d-PGJ_2_), can protect endothelial cells from ROS through the induction of GSH production and cytoprotective genes such as heme oxygenase expression [17,45,46]. In contrast, high intracellular concentrations of electrophilic molecules can interact with nucleophilic targets in uncatalyzed reactions [19,47]. The lipid electrophile, HNE, is found in high concentrations in Alzheimer’s disease and is believed to promote protofibril formation [48]. Elegant work by Codreanu et al. explored functional protein systems most susceptible to alkylation by electrophilic species [49]. Inflammation and degenerative pathologies can trigger oxidative stress that generates lipid electrophiles capable of modifying proteins, triggering alternative cell signaling and ultimately engaging cell death [30,50]. 

## 3. Metabolism and Inflammation

**Figure 3:**
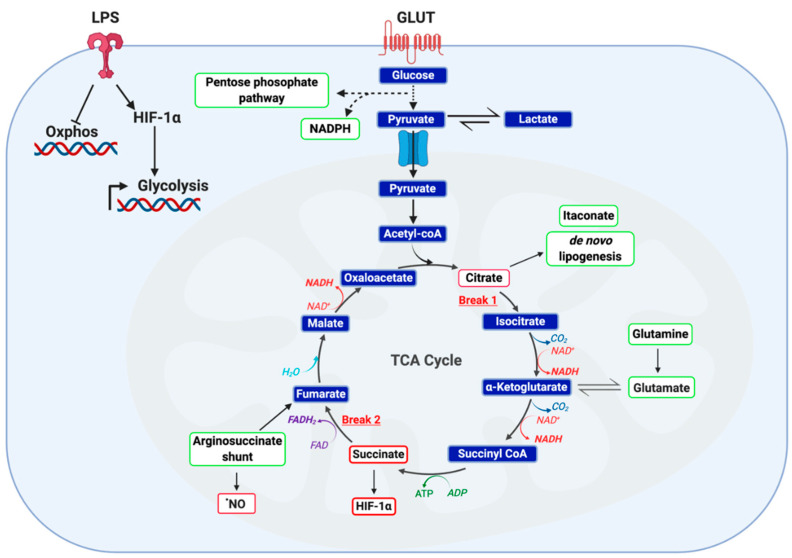
Metabolic phenotype of classically LPS-activated macrophage. LPS activated macrophages are defined by a highly glycolytic phenotype with a disrupted tricarboxylic acid (TCA) cycle and attenuated cellular respiration. Recent studies employing carbon flux analysis identified two breaks in the TCA cycle, one at isocitrate dehydrogenase (IDH) and the other at succinate dehydrogenase (SDH). This phenomenon results in the accumulation of both citrate and succinate, key metabolic intermediates capable of functioning as inflammatory signaling mediators. While citrate functions as a precursor to itaconate, prostaglandins and ^•^NO, succinate is capable of protein post-translational modification and stabilizing HIF-1α. Furthermore, accumulation of both succinate and reactive oxygen species (ROS) stabilize HIF-1α, thereby potentiating glucose utilization and downstream inflammatory programming. It has been posited that succinate accumulation in LPS-activated macrophages is the result of itaconate production and subsequent inhibition of SDH) Finally, two anaplerotic pathways are utilized by activated macrophage as a fuel source: the glutamine shunt and the arginosuccinate shunt. Together, these changes manifest in altered cellular metabolism to meet the bioenergetic demands of the cell.

Changes in intracellular metabolic pathways can dictate immune cell function. For several decades, it has been recognized that immune cells have unique bioenergetic demands required for effector function. Enhanced glycolytic activity, as an example, is considered to be a canonical feature of the rapid activation of immune cells, such as macrophages and dendritic cells. Alterations across metabolic pathways are responsible, in part, for effector cytokine production by CD8^+^ T cells, phagocytosis by macrophage, and antigen presentation by dendritic cells [51,52]. The canonical metabolic changes in LPS-activated macrophages, for example, are depicted in Figure 3. Metabolism is central to the evolution of the immune response. In recent years, immunometabolism has become a therapeutic target for the treatment of inflammatory pathologies. The subsequent section focuses on transcription factors known to interact with metabolic pathways that are targeted by electrophilic compounds.

### 3.1. NRF2

**Figure 4:**
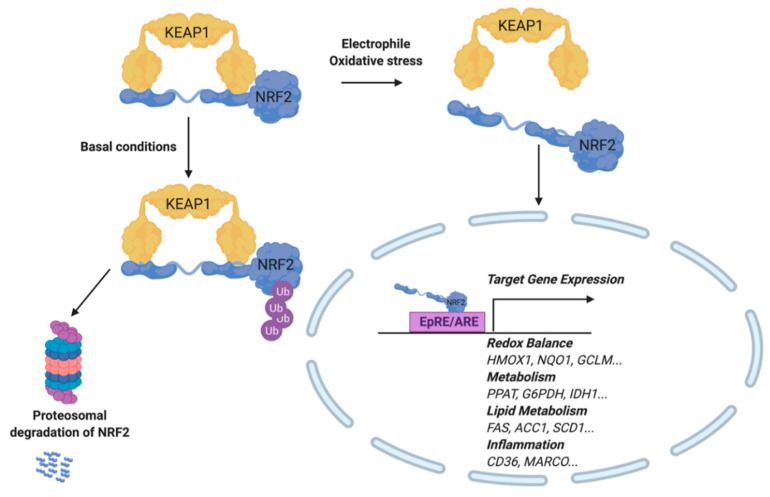
Activation of the Electrophilic Response Element/Antioxidant Response Element (EpRE/ARE). During oxidative stress or exogenous addition of an electrophile, NRF2 is released from its cytosolic binding partner, KEAP1. NRF2 translocates to the nucleus, where it binds to the Antioxidant Response Element (ARE) and induces target gene expression. Target genes include those involved in redox balance, metabolism, lipid metabolism, and inflammation. Under basal conditions, NRF2 remains bound to KEAP1, while subsequently experiencing ubiquitinylation and proteasomal degradation.

The transcription factor NRF2 regulates the gene product expression of the EpRE/ARE. Activity of this transcription factor is dependent on the redox-sensitive inhibitor, Kelch-like ECH-associated protein 1 (KEAP1). More specifically, KEAP1 binds directly to NRF2 and recruits CUL3 (an E3 ubiquitin ligase) to target NRF2 for proteasomal degradation. Upon Michael addition of KEAP1 with an electrophile at reactive cysteines, NRF2 is capable of translocating to the nucleus and activating EpRE-dependent genes (Figure 4) [53]. Interestingly, both electrophiles and RS are capable of modifying KEAP1 to stabilize newly synthesized NRF2 [54,55]. While KEAP1 contains 27 cysteines, not all are reactive, and each has a different reactivity as well as specificity for electrophilic species. For example, the electrophilic fatty acid nitro-oleic acid (NO_2_-OA) activates NRF2 by adduction at KEAP1 Cys^38^, Cys^226^, Cys^257^, Cys^273^, Cys^288^, and Cys^489^. Interestingly, NRF2 activation by this compound occurred by way of a Cys^151^-independent mechanism while Cys^273^ and Cys^288^ accounted for nearly 50% of KEAP1-NO_2_-OA interactions [56]. In contrast, sulforaphane activates NRF2 most commonly through KEAP1 adduction at Cys^151^ [57]. 

NRF2 activity has been identified as a key regulator responding to environmental stimuli. Not surprisingly, the NRF2 regulatory network extends to altering cellular bioenergetics and intermediary metabolism. This is illustrated most clearly by the NRF2-mediated transcription of genes involved in the pentose phosphate pathway (PPP) including: glucose 6 phosphate dehydrogenase; phosphogluconate dehydrogenase, malic enzyme 1, isocitrate dehydrogenase 1, transketolase, and transaldolase [58,59,60,61,62]. The PPP is essential for generating equivalents of NADPH, a reducing agent used in anabolic reactions and ROS essential to immune cell activation. Additionally, NRF2 has been shown to regulate expression of genes involved in purine nucleotide synthesis (phosphoribosyl pyrophosphate amidotransferase, methylenetetrahydrofolate dehydrogenase 2) [59,63]. 

Well known as a redox-sensitive transcription factor, NRF2 has also been shown to modulate metabolism in pathologies characterized by unattenuated inflammation. In the setting of metabolic syndrome, a chronic inflammatory state, a high-fat diet was sufficient to repress *NRF2* expression. Moreover, this reduction in *NRF2* expression was accompanied by an increase in hepatic and serum cholesterol as well as free fatty acids [64]. Intermediary metabolism may further be affected by NRF2 through the modulation of enzyme activity of proteins with susceptible thiols that are normally affected by ROS and RNS [63]. 

Emerging evidence suggests that these RS influence the redox modeling of effector proteins and gene programs that regulate the immune response. For example, studies have suggested that T cells, in vitro and in vivo, require mROS generated from complex III in order to activate Nuclear Factor of Activated T-cells (NFAT) and produce interleukin-2 (IL-2). The investigators suggested that this effect may be attributed to ROS-dependent perturbations in redox-sensitive signaling kinases. Interestingly, transcriptional programs such as Nrf2/Keap1, NF-κB, members of the AP-1 family and, HIF-1α are all modulated by ROS-dependent redox signaling. HIF-1α is directly targeted by ROS as well as indirectly impacted through redox modulation of prolyl hydroxylases. HIF-1α has been implicated in the metabolic reprogramming of activated macrophage, dendritic cells, and T cells [65,66,67]. 

The antioxidant response gene products downstream of NRF2 may prevent oxidation of susceptible Cys or reverse this effect through the reduction of these same moieties. For example, pyruvate kinase (PK) activity is inhibited by ROS oxidation at Cys^358^ or Cys^436^. Depending on the isoenzyme, this results in the accumulation of glycolytic intermediates and engagement of the pentose phosphate pathway [68,69]. It would be expected that NRF2 activation would increase the activity of PK through detoxification of ROS. However, studies have shown that NRF2 actually reduces the expression of PK [70]. 

In addition to the susceptibility of glycolytic enzymes, mitochondrial enzymes involved in both fatty acid oxidation and the tricarboxylic acid cycle (TCA) cycle have Cys residues susceptible to oxidation. Enzymes involved in β-oxidation as well as pyruvate dehydrogenase kinase 2 are susceptible to oxidation and alkylation at cysteines [71,72]. It is possible, therefore, that the electrophilic induction of NRF2 activity would antagonize the ROS-induced inhibition of β-oxidation and carbon flux into the TCA [73]. Finally, previous studies have demonstrated that AMP-activated protein kinase (AMPK), a central node in nutrient sensing and regulation, can be oxidized. The effect of RS on AMPK activity is not fully elucidated. However, as the master regulator of the EpRE/ARE, it would be hypothesized that Nrf2 would inhibit AMPK oxidation by ROS [74]. 

### 3.2. NF-κB

While electrophiles have historically been recognized for their engagement and potentiation of the EpRE/ARE, these molecules can also modulate inflammation as pleiotropic cell signaling mediators [17]. In addition to interfacing with nuclear transcription factors, heat shock protein response and apoptosis pathways, electrophiles can also modulate inflammation directly through the NF-κB pathway [75]. 

**Figure 5:**
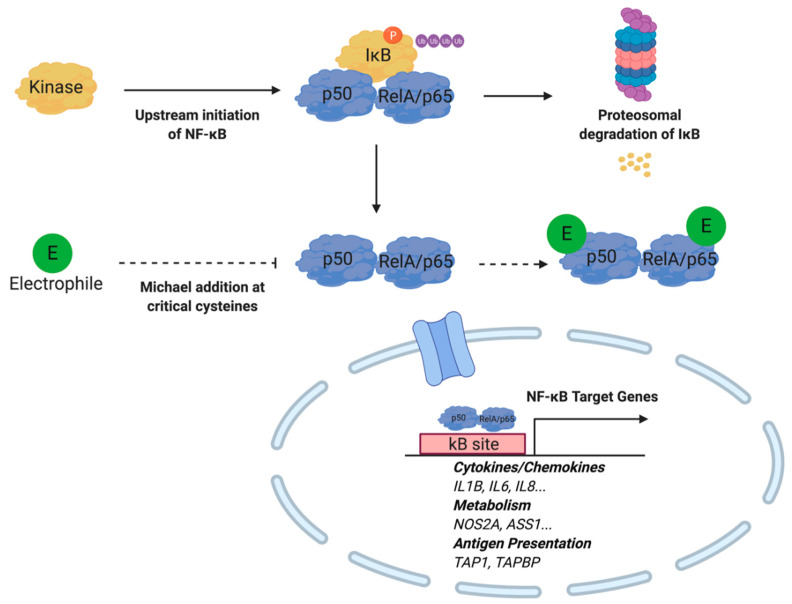
Electrophiles inhibit NF-κB activity by binding to the p50 and RelA/p65 protein subunits. Michael addition at cysteine moieties prevent nuclear translocation required for target gene expression. Under inflammatory conditions, kinase activity induced by upstream NF-κB signaling results in phosphorylation, ubiquitinylation, and proteasomal degradation of IκB. Subsequently, NF-κB translocates to the nucleus to initiate target gene expression.

Previous studies have demonstrated that electrophiles are capable of regulating NF-κB activity at multiple nodes within the signaling cascade. For example, nitrated fatty acids (NO_2_-FA) inhibit NF-κB-dependent transcription in LPS-activated RAW264.7 cells as measured by a NF-κB-luciferase reporter construct. Subsequent analysis utilizing immunoprecipitation techniques illustrated that NO_2_-FAs covalently bind to the p65 subunit of the inflammatory transcription factor at multiple Cys [76]. In addition to the direct modulation of NF-κB, the cyclopentenone neuroprostane, A4-NP, indirectly promotes the degradation of this transcription factor. It is believed that A4-NP covalently binds to the Cys^179^ of the inhibitor of NF-κB kinase (IKK), thereby inhibiting the phosphorylation and degradation of Iκβ, which normally interacts with the p65 unit of NF-κB to prevent the transcription factor’s nuclear translocation [29]. Cyclopentanone prostaglandins such as 15d-PGJ2 inhibit NF-κB signaling through Michael adduction at critical cysteine residues on the DNA-binding domain of this transcription factor and IκB kinase [77]. Similarly, the lipid electrophiles 15-oxoeicosatetraenonic acid (15-oxo-ETE) and nitro-oleic acid NO_2_-OA are capable of inhibiting NF-κB signaling through IKKβ [78,79]. A general outline of electrophile regulation of NF-κB signaling is depicted in Figure 5. The anti-inflammatory activity of lipid electrophiles extends beyond inflammatory pathologies. Interestingly, in a model of xenograft triple negative breast cancer, NO_2_-OA treatment was sufficient to inhibit tumor growth while increasing ubiquitination of NF-κB [80]. 

Downstream effects of the electrophilic inhibition of NF-κB have revealed the suppression of canonical inflammatory mediators. For example, both NO_2_-FA and A_4_-NP have been shown to inhibit the production of cytokines tumor necrosis factor (TNF)α and interleukin (IL)-1β in vitro [29,81]. The anti-inflammatory downstream effects of electrophile activity extend beyond cytokine production. NF-κB inhibition leads to an observed reduction in both COX-2 and iNOS mRNA and protein expression [29]. Both of these enzymes are intimately linked to both the inflammatory response and cellular metabolism. COX enzymes are responsible for converting arachidonic acid into prostaglandins, most notably prostaglandin E_2_ (PGE_2_). PGE_2_ and hydroxyl metabolites, resulting from the oxidation of arachidonic acid or docosahexaenoic acid, may be further oxidized by 15-hydroxyprostaglandin dehydrogenase and other oxidoreductases to form electrophilic oxo-fatty acids [40,78,82]. Interestingly, PGE_2_ was found to have a profound effect on dendritic cell maturation and dendritic cell-directed T-cell differentiation [83,84,85]. The oxo-fatty acid class of molecules, including 15-oxo-ETE and 17-oxo-DHA, has demonstrated the ability to inhibit NF-κB signaling [78,86]. More specifically, previous work by Cipollina et al. suggests that 17-oxo-DHA further modulates inflammation by preventing the release of mature IL-1β through the inhibition of the NLRP3 inflammasome [87]. 

The electrophilic inhibition of iNOS protein expression also impacts immune cell effector function and metabolism. While required for the oxidative burst in innate immune cells, **^•^**NO has also been shown to determine complex I abundance in inflammatory macrophage and modulate the relative abundance of citrate, itaconate, and succinate [88]. Through both direct and indirect regulation of the inflammatory program, electrophiles are capable of modulating immune cell metabolism through NF-κB. 

### 3.3. PPARγ 

In addition to altering the redox balance of the cell, NRF2 further regulates metabolism through modulation of Peroxisome Proliferator-Activated Receptor Gamma (PPARγ activity. PPARγ forms a heterodimer with the retinoid X receptor (RXR) and has been shown to regulate the expression of genes involved in glucose metabolism, lipid metabolism, adipogenesis, and immune responses [89,90,91,92]. Interestingly, PPARγ activity is regulated by electrophiles at the level of gene expression and protein activity. More specifically, the *PPARG* gene promoter contains an EpRE/ARE that may be activated upon electrophile engagement of the Nrf2-Keap1 system [93,94]. The gene product is further regulated at the ligand binding-domain of the C-terminal region. This large hydrophobic domain is capable of binding both long-chain and electrophilic fatty acids [95,96]. Michael addition at Cys^285^ by electrophilic lipids such as 15d-PGJ_2_, 4-oxo-DHA, 5-oxo-EPA, 6-oxo-ETE, arachidonic acid derivatives, and nitroalkenes have been reported to activate PPARγ activity [97,98,99]. 

**Figure 6:**
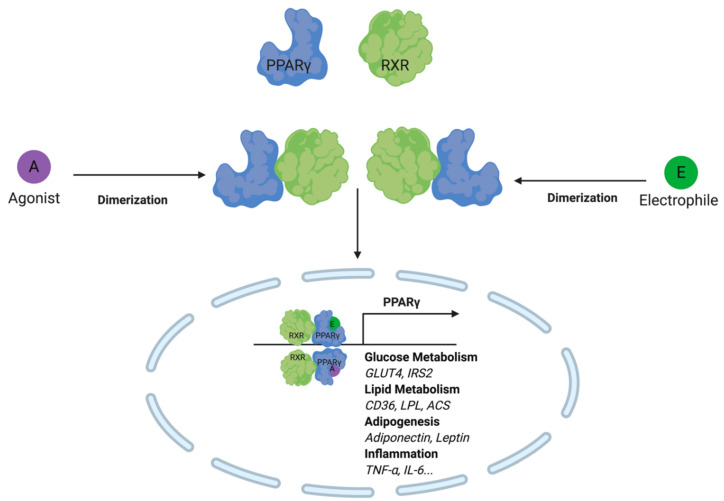
Lipid electrophiles activate PPARγ by creating an adduct at the c-terminal region ligand-binding domain. Similar to PPARγ agonists, lipid electrophiles activate the transcription factor and trigger target gene expression. Target genes include those involved in glucose metabolism, lipid metabolism, adipogenesis and inflammation.

Upon adduction, PPARγ undergoes a conformational shift that results in co-regulatory protein recruitment and downstream protein expression of genes involved in lipid metabolism (Figure 6). Macrophage, more specifically, clear lipids through PPARγ activation of CD36, liver X receptor (LXR), and ABC transporters [100]. The effect of this transcription factor on lipid metabolism has important downstream implications for myeloid cell immune function. For example, in developing human dendritic cells, some of the most significant changes in gene expression were those involved in lipid metabolism. In contrast, genes linked to immune response and effector function changed most significantly later on in cell maturation [101]. In other bodies of work, PPARγ has been shown to directly influence immune cell effector function through antigen presentation. More specifically, through the modulation of retinoid metabolism and retinoic acid receptor (RAR)/RXR signaling, CD1 expression is altered, thereby influencing the dendritic cells’ ability to cross-present lipid antigens to lymphocytes [102,103]. 

When macrophages are activated by interleukin-4, signal transducer and activator of transcription 6 (STAT6) as well as PPARγ-coactivator-1β (PGC-1β) activate mitochondrial biogenesis and increase fatty acid oxidation. Interestingly, investigators discovered that expression of PGC-1β in a transgenic mouse model was sufficient to suppress pro-inflammatory cytokine expression. In contrast, the pharmacological inhibition of fatty acid oxidation with etomoxir and RNAi knockdown of PGC-1β both attenuated arginase activity—a marker of alternative macrophage activation [104]. This study effectively establishes a link between mitochondrial metabolism and macrophage activation through the PPARγ program. 

One study has linked PPARγ activity to autoimmunity through negative regulation of T-cell activation. In CD4^+^-PPARγ^KO^ mice, there was an identified increase in activated T cells, follicular helper T cells, and germinal center B cells. This change in immune cell phenotype was accompanied by an increase in auto-antibody production [105]. While this transcription factor has been implicated in modulation of the adaptive immune response, PPARγ has also been shown to alternatively activate macrophage. Odegaarden et al. demonstrated that macrophage-specific deletion of this transcription factor predisposed mice to diet-induced obesity, insulin resistance, as well as glucose intolerance [106]. 

## 4. Electrophiles as Potential Therapeutics

**Figure 7:**
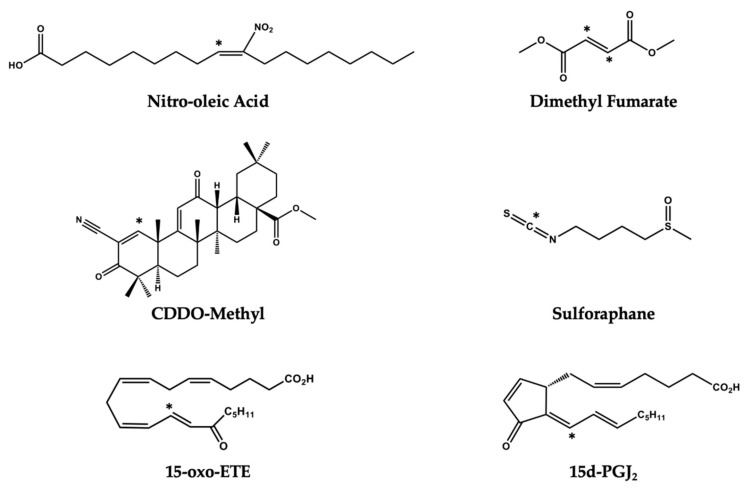
Electrophilic molecules with therapeutic application. Electrophilic carbons on each molecule are denoted with an asterisk (*).

While the formation and signaling actions of pro-inflammatory lipid signaling mediators have been identified as therapeutic targets, there are fewer studies focused on harnessing endogenously formed lipid electrophiles that promote resolution and inhibition of inflammation [107,108,109]. Electrophilic molecules represent a promising class of drug candidates that modulate cellular homeostasis through their pleiotropic effect as cell signaling mediators. Initial interest in exploring these bioactive molecules stems from the fact that electrophiles have been detected at notable concentrations in a number of pathologies. For example, cyclopentenone neuroprostanes have been measured in the frontal cortex of patients with Alzheimer’s disease, oxo-ETEs have been identified in the lungs of patients with pulmonary hypertension, and nitroalkenes have been identified in the mitochondria of cardiomyocytes following ischemia reperfusion. Each of these molecules demonstrates anti-inflammatory effects and was, therefore, initially hypothesized to be protective in disease states. Endogenously formed electrophiles such as oxo-fatty acids and NO2-FA, as well as already approved therapeutics such as Dimethyl Fumarate (DMF), Bardoxolone (CDDO-Me), and sulforaphane (SFN), represent promising drug candidates with growing evidence indicating their ability to treat inflammatory pathologies through modulating immune cell metabolism (Figure 7). Recently, itaconate has been identified as a macrophage metabolite capable of modulating immune cell function. Itaconate generated through the enzyme encoded by *Irg1* accumulates upon macrophage activation with lipopolysaccharide. While functioning as a weak electrophilic substrate, itaconate is believed to exert its anti-inflammatory effect through direct inhibition of the TCA enzyme succinate dehydrogenase [110]. Moreover, itaconate is believed to exert its anti-inflammatory effect in part through the activation of the EpRE/ARE. This metabolite activates antioxidant and anti-inflammatory effects through alkylation of Cys^151^, Cys^257^, Cys^273^, Cys^288^, and Cys^297^ on the KEAP1 protein [111]. However, these studies were conducted with the use of the itaconate surrogate, octyl-itaconate and it has yet to be shown that endogenous levels of itaconate, which reach millimolar concentrations, are capable of PTM of KEAP1 in vivo. While octyl-itaconate may have different targets than endogenous itaconate, this modified, electrophilic, immune-metabolite has demonstrated clear anti-inflammatory properties and warrants further investigation as a potential therapeutic.

Interestingly, itaconate-dependent activation of NRF2 was sufficient to inhibit IL-1β transcription in the context of LPS-activated macrophages. Hooftman et al. expanded upon this important body of work by characterizing the effect that this immunomodulatory metabolite has on inflammasome-dependent IL-1β production and maturation [112]. More specifically, itaconate was found to alkylate Cys^548^ on NLRP3, thereby inhibiting the interaction between this protein and NEK7, a partner required for inflammasome activation. Additional research will be needed to evaluate if itaconate can be used as a therapeutic to treat NLRP3-driven inflammatory pathologies [111,112]. 

Methylglyoxal, another endogenous electrophilic metabolite, has demonstrated a similar ability to activate NRF2-KEAP1 signaling. Inhibition of the glycolytic enzyme PGK1 is shown to accumulate methylglyoxal, which subsequently modifies the KEAP1 protein and activates the NRF2-dependent transcriptional program [113]. Additionally, Gaffney et al. demonstrated that methylglyoxal can alter metabolism through the PTM of glycolytic enzymes. More specifically, the authors of this paper demonstrated that methylglyoxal rapidly conjugates with glutathione via the enzyme glyoxalase 1, generating lactoylglutathione (LGSH). LGSH can subsequently be hydrolyzed by glyoxalase 2 to cycle glutathione and generate lactate. The investigators identified a non-enzymatic acyl transfer of lactate from LGSH to protein Lys residues of glycolytic enzymes. This “LactoylLys” modification of metabolic proteins characterizes a previously unexplored feedback mechanism for regulation of central metabolism [114]. These studies highlight the interplay between endogenously formed electrophilic metabolites and metabolic regulation of the NRF2-KEAP1 signaling in the EpRE/ARE.

### 4.1. Lipid Electrophiles

Polyunsaturated fatty acids (PUFAs) are ideal substrates for generating lipid electrophiles. These molecules form in oxidative environments, such as those created by an inflammatory response, and function to regulate subsequent cell signaling events through PTM [16]. Lipid electrophiles can form through enzymatic or non-enzymatic reactions with precursor molecules harnessed directly from dietary sources or formed from the metabolite of a xenobiotic. Lipid electrophiles include oxo-fatty acids, nitro-fatty acids, cyclopentenone prostaglandins and neuroprostanes, keto prostaglandins, and electrophilic fatty acid metabolites such as HNE and ONE. The diverse anti-inflammatory and metabolic effect of these pleiotropic signaling molecules are illustrated by oxo-fatty acids such as 15-oxo-ETE and 17-oxo-DHA. For example, both molecules have a demonstrated the ability to activate the EpRE/ARE, inhibit NF-κB signaling, and activate PPARγ [78,86,87,97,99]. The nitro-fatty acid, NO_2_-OA, is similarly capable of Nrf2 activation, PPARγ partial agonism, activation of heat shock proteins as well as inhibition of TLR4/NF-κB/STAT1 signaling [115,116,117]. 

Mitochondria have been identified as an ideal subcellular location for the generation of lipid electrophiles. As a source of both unsaturated fatty acids and oxidizing agents, the mitochondria produce signaling mediators such as nitro- and oxo-fatty acids during inflammatory responses. It is well documented that inflammation increases concentrations of O_2_**^•^**^−^, H_2_O_2_, **^•^**NO and **^•^**NO_2_, thereby promoting lipid electrophile generation [17,118]. 

Lipid electrophiles are capable of influencing cellular homeostasis through the regulation of cell signaling by way of PTM or adjusting the redox state of the cell. As outlined by Schopfer et al., the mitochondrion is well positioned for reactions with electrophilic fatty acids for a number of reasons: (1) A number of mitochondrial proteins have been identified as having electrophile reactive cysteines [119]. (2) The matrix pH changes during cellular respiration, yielding more reactive thiol groups capable of undergoing Michael addition [119]. (3) Sulfhydryl groups contained within the lipoic acid cofactor on NADH generating enzymes α-ketoglutarate dehydrogenase (αKGDH) and pyruvate dehydrogenase (PDH). (4) Fatty acid electrophiles uncouple respiratory chain function, alter OXPHOS, and thereby reduce ROS production [16,71,120,121]. As mentioned above, the peroxisome functions as a subcellular location for fatty acid metabolism and redox modulation. Recent studies have demonstrated that RS are produced and processed by the peroxisome, making this organelle a point of focus for redox balance [122,123]. Moreover, as a subcellular location for polyunsaturated fatty acid metabolism and catabolism, the peroxisome generates the structural backbone for lipid molecules that attenuate inflammation [124,125]. Given the growing appreciation for how RS influence immune cell metabolism and the importance of endogenous lipid mediators of inflammation, the peroxisome represents a node for immune cell regulation [123].

Tissue glycolytic rates are closely linked to the redox state of the cell [126,127]. Electrophilic PTM of glycolytic enzymes provides a rapid response mechanism to the metabolic demands of the cell. In addition to the phosphorylation-dependent cell signaling, many of these key enzymes can be regulated through their nucleophilic cysteine and histidine residues. Michael addition at these redox-sensitive amino acids can effectively alter enzyme activity. For example, the lipid electrophile HNE adducts to glucose transporter 3 (GLUT3) and thereby attenuates glucose influx [75,128]. Additionally, 15d-PGJ_2_ will adduct to both enolase and lactate dehydrogenase (LDH), thereby inhibiting the activity of both enzymes [75,129]. A list of electrophilic molecules that have been shown to interact with glycolytic enzymes, as well as other proteins directly involved in metabolism, is shown in Table 1 [130,131,132,133,134]. 

It has previously been demonstrated that NO_2_-OA inhibits the expression of iNOS and subsequent **^•^**NO production in activated macrophages. Unpublished research from our laboratory investigated whether the inhibition of **^•^**NO production is sufficient to explain the changes observed in LPS-activated murine macrophage treated with NO_2_-OA. Preliminary data reveals that there is a difference in the metabolic profile of activated macrophage treated with NO_2_-OA as compared to macrophage treated with 1400 W, a selective inhibitor of iNOS. This finding suggests to us that the effects of NO_2_-OA on macrophage polarization after LPS activation are not only due to the inhibition of iNOS, but also due to the pleiotropic signaling and alteration of pathways involved in metabolism.

### 4.2. Dimethyl Fumarate

DMF is currently used to treat relapsing multiple sclerosis (MS). As a methyl ester of fumaric acid, when internalized by the cell, the molecule is rapidly hydrolyzed to its active metabolite, monomethylfumarate [136,137]. Interestingly, DMF is an analogue of the TCA metabolite, fumarate. This electrophile has been shown to reduce relapse rate and time to progression in phase III clinical trials for patients with MS [138]. Fumaric acid is the electrophilic subcomponent of DMF and has also been used to treat inflammatory conditions like psoriasis [135]. The mechanism underlying DMF therapeutic efficacy is still being elucidated. However, as a pleiotropic electrophile, this drug has demonstrated the ability to modulate immune cell metabolism at a number of nodes.

In addition to engaging the EpRE/ARE through Nrf2-Keap1 signaling, DMF has also been shown to adduct to the glycolytic enzyme, GAPDH. This interaction inhibits glycolysis and limits immune cell activation [139]. Using a chemical proteomic platform, Blewett et al. identified cysteines sensitive to DMF and linked to T-cell activation. This study identified that DMF blocked the association of PKCθ with the co-stimulatory receptor CD28 [140]. More specifically, T-cell activation is inhibited by limiting subsequent CD28 kinase signaling through DMF adduction to PKCθ cysteine residues [140]. Additionally, DMF inhibits IRAK4-MyD88 interaction by adducting to Cys^13^ of IRAK4 in human innate immune cells [141]. Inhibition of NF-κB activation by way of IRAK4 limits **^•^**NO production by iNOS. This has direct implications for cellular metabolism as **^•^**NO would otherwise limit the respiratory capacity of the cell. At a macro level, changes in global metabolism have also been documented in patients actively taking DMF as an increase in lipid metabolism [142]. 

The aforementioned changes in immune cell metabolism have accompanied documented changes in cell function. Indeed, DMF has been shown to attenuate innate immune cell activation and polarization both in vitro and in vivo. In a mouse model of experimental allergic encephalomyelitis (EAE), DMF significantly reduced the number of Mac-3 positive microglia and macrophages [143]. Mononuclear phagocytes treated with this electrophile in vitro demonstrated a shift from a M1-like polarization to an M2-like polarization [144]. The functional implications of this treatment have been evinced by the reduction in CXCL8, CXCL-9, and CXCL-10 chemokines in both LPS and INF-γ-treated peripheral blood mononuclear cells (PBMCs) [144]. In addition to attenuating inflammation in mononuclear cells, DMF has also been shown to inhibit DC maturation [145]. 

Similarly, lymphocytes within circulation are influenced by DMF treatment. More specifically, this electrophile demonstrates the ability to shift cytokine production from a Th1-like to a Th2-like profile [146]. Patients who have MS and received 12 months of DMF treatment displayed an increase in monocytes as well as naïve T and B cells. In contrast, the percentages of effector memory T cells and CD4^+^ T cells expressing IFN-γ, GM-CSF, and IL-17 decreased [147]. 

### 4.3. Bardoxolone

CDDO-Me, also known as BAR, RTA402, or CDDO, is a synthetic triterpenoid currently under clinical investigation for treatment of chronic kidney disease and pulmonary hypertension. This electrophile has documented anti-inflammatory, anti-fibrotic and anti-proliferative effects that have led to significant interest in pre-clinical investigations exploring treatment of pathologies with immunological components [148,149,150]. 

Similar to other bioactive electrophiles, CDDO-Me has a pleiotropic effect on activated immune cells. CDDO-Me activation of the EpRE/ARE regulates the redox balance and, subsequently, the bioenergetic demands of the cells. In addition, CDDO-Me has demonstrated the ability to bind to the ATP-dependent molecular chaperon, HSP90 [151]. This interaction inhibits HSP90 clients, including elements of cell signaling cascades linked to cellular nutrient utilization such as EGFR, ErbB2, mTOR, and STAT3 [152,153]. 

CDDO-Me has also demonstrated efficacy as a potential immune-modulating anti-cancer agent. In an animal model of estrogen receptor negative (ER^−^) breast cancer, treatment with this electrophile was sufficient to delay tumorigenesis and inhibit tumor-associated macrophage (TAM) infiltration of solid tumors [154]. Further studies published by Ball et al. determined that CDDO-Me was also capable of transitioning macrophage polarization from M2 to M1 [155]. It is important to recognize that this observed macrophage polarization reflects an inflammatory immune cell profile that is beneficial in the context of tumor immunity, but would be detrimental in the setting of autoimmune pathology. Interestingly, CDDO-Me treatment led to significant reduction in intratumoral regulatory T cells but a significant increase in CD8^+^ T cells [156]. This finding echoes a larger body of research indicating that improved immune cell penetration of the tumor microenvironment can be accompanied by increased anti-tumor activity [157,158]. 

It is well accepted in cancer research that metabolism regulates tumor-associated immune cell activation and polarization [159,160,161]. It is highly likely, therefore, that the observed changes in the immune profile of CDDO-Me-treated tumors are mediated in part through altering cellular metabolism and therefore tumor-associated immune cell function.

### 4.4. Sulforaphane 

SFN is an isothiocyanate, a class of phytochemical electrophiles produced by cruciferous vegetables [162,163]. Members of the isothiocyanate family signal through the Nrf2-Keap1 pathway, targeting Cys^151^ in the KEAP1 BTB domain [17]. In recent years, pre-clinical studies using SFN have focused on the compound’s potential as a treatment for metabolic syndrome or malignancy [164,165]. In the context of insulin resistance and cancer, SFN activates the EpRE/ARE to address inflammation. Interestingly, one study indicated that T cells treated with SFN demonstrated a reduction in the expression of activation markers while also inhibiting cell proliferation [166]. SFN treatment in an experimental model of autoimmune encephalomyelitis was sufficient to protect against T-cell-mediated disease progression through the inhibition of IL-23 and IL-12 cytokine production in dendritic cells [167]. 

The wide-ranging metabolic effects of SFN can be attributed the molecules’ activation of the insulin receptor substrate-1/protein kinase B (IRS-1/PKB) signaling cascade. Phenotypically, this electrophile has been shown to reduce ceramide production, improve glycogenolysis and inhibit gluconeogenesis. Hepatocytes treated with SFN, for example, demonstrate the anti-steatotic effects of this electrophile [168,169]. In the context of carcinogenesis, SFN induces an anti-proliferative effect and alters the immunologic tumor microenvironment. The anti-proliferative activity of SFN is mediated through inhibition of fatty acid metabolism and glucose metabolism. In a murine model of prostate cancer, SFN decreased protein and mRNA levels for CPT1A, the enzyme responsible for fatty acid uptake and subsequent β-oxidation. This change in protein expression was accompanied by a significant decrease in plasma and prostate adenocarcinoma levels of free fatty acids, total phospholipids, acetyl-CoA and ATP upon treatment with SFN [170]. A separate study found that SFN induced apoptosis in the prostate cancer cell line, LNCap. Interestingly, the study suggests that cell death is mediated by drug induced reduction in phosphoglucomutase 3 (PGM3), as SFN treatment lead to a significant reduction in expression of this metabolic enzyme. Considering the effect of SFN on cancer cell viability, this electrophile may mediate anti-proliferative activity by regulation of cancer cell metabolism [171]. 

In addition to studies examining the effect of SFN directly on cancer cells, recent work has also documented the effect of this electrophile on tumor-associated immune cells. Kumar et al. identified that SFN treatment was sufficient to reduce the number of monocyte myeloid derived suppressor cells (mMDSCs) and increase the number of mature dendritic cells for monocytes exposed to glioma conditioned media [172]. This finding has potential therapeutic implications as glioma avoidance of immunosurveillance is mediated by MDSC inhibition of T-cell proliferation [173]. It is well established that metabolic reprogramming is essential to the maturation of the immune response [174,175]. In fact, previous studies have demonstrated that dendritic cell maturation and activation is regulated by changes in metabolism [176,177]. It is, therefore, quite interesting that the immunosuppressive effector function of MDSC, a cell line that inhibits maturation of the immune response, is linked to lipid metabolism [178]. Given that SFN modulates immune cell maturation in glioma condition media, it is possible that the pleotropic signaling activity of this electrophile mediated this change through modulation of metabolism. 

## 5. Conclusions

Metabolism regulates the maturation and effector function of immune cells. During inflammatory states, the cell may experience an altered redox potential. The RS produced during both acute and chronic inflammatory conditions are sufficient to alter cell signaling through the oxidation of lipids, amino acids, and even nucleic acids. By altering signaling cascades, RS effectively dictate the propagation of the inflammatory sequence. Electrophilic compounds represent promising pharmacotherapies with the ability to modulate inflammation through a two-hit approach: (1) functioning as pleiotropic cell signaling mediator through PTM of key redox regulatory proteins and (2) modulating immune cell metabolism. Electrophiles represent ideal therapeutic candidates for the treatment of inflammatory conditions because they are capable of inhibiting NF-κB pro-inflammatory signaling and activating the Nrf2-Keap1 antioxidant signaling pathway while also functioning as reversibly reactive signaling mediators. There is mounting evidence demonstrating that the redox potential of the cell is linked to metabolic signaling, especially signaling through susceptible cysteines within metabolic enzymes. It is important, therefore, that future studies employing chemical proteomic techniques are used to identify the cysteine residues on metabolic enzymes capable of adducting with electrophiles. Previously published work has identified, for example, electrophilic Michael addition of enolase, lactate dehydrogenase, glyceraldehyde 3-phosphate dehydrogenase as well as glucose transporters. However, specific studies focused on identifying protein adducts within each class of immune cells at each stage of maturation are needed to create targeted therapies with electrophilic drug candidates. Due to the high concentration of electrophilic compounds typically used for in vitro studies, it will be essential that investigators validate these discoveries in vivo to assess the clinical viability for novel, electrophilic therapeutic candidates. 

## Figures and Tables

**Table 1:** A list of electrophilic molecules that have been shown to interact with glycolytic enzymes, as well as other proteins directly involved in metabolism.

Protein	Electrophile	Residue	Ref
GAPDH	NO_2_-OA	C-153, C-244, H-108, H-134, H-327	[130]
	DMF	C-152, C-156, C-247	[135]
	HNE	C-244, C-281, H-327, H-164, K-331	[131]
	4-OI	C-22	[134]
Aldolase	HNE	H-246	[132]
Enolase	HNE	Not well-defined	[75,129]
GLUT3	HNE	Not well-defined	[128]
LDH	15-d-PGJ_2_	Not well-defined	[75]
H-Ras	15-d-PGJ_2_	C-184	[132]
Erk-2	HNE	H-178	[133]
α-KGDH	HNE	Lipoic acid sulfhydryl	[71]
PDH	HNE	Lipoic acid sulfhydryl	[71]
